# Factors associated with anxious distress in major depressive episodes: a cross-sectional study

**DOI:** 10.1192/j.eurpsy.2024.628

**Published:** 2024-08-27

**Authors:** F. Bartoli, B. Bachi, T. Callovini, D. Palpella, S. Piacenti, M. Morreale, M. E. Di Lella, C. Crocamo, G. Carrà

**Affiliations:** ^1^Department of Medicine and Surgery, University of Milano-Bicocca , Monza, Italy; ^2^Division of Psychiatry, University College London, London, United Kingdom

## Abstract

**Introduction:**

The comorbidity between depression and anxiety is a common occurrence. The DSM-5 introduced the “anxious distress” (AD) specifier that can be applied to any depressive episode – both in major depressive disorder (MDD) and bipolar disorder (BD) – when symptoms such as feelings of tension, restlessness, difficulty concentrating, and fear that something awful may happen or to lose control are present. Longitudinal data showed that the AD specifier may be an effective predictor of chronicity, time to remission, and functional disability in depressive disorders. In addition, evidence on AD proved its association with increased depressive symptom severity.

**Objectives:**

Available literature seems to suggest that AD occurs in a specific subgroup of patients, thus enabling a peculiar clinical profile to be outlined. To expand knowledge in this field, we performed a cross-sectional study aimed at identifying clinical correlates of AD in people with major depressive episodes.

**Methods:**

Adult people admitted to two psychiatric inpatient units in the northern area of the Metropolitan City of Milan from May 2020 to December 2022 were screened for a major depressive episode and relevant specifiers using the Structured Clinical Interview for DSM-5 (SCID-5). Data on socio-demographic and clinical variables were collected. The severity of depressive and manic symptoms was assessed using the Montgomery–Åsberg Depression Rating Scale (MADRS) and Young Mania Rating Scale (YMRS), respectively. Univariate comparisons between participants with and without AD were conducted, and two multiple logistic regression models were arranged to investigate the association between AD and candidate explanatory variables.

**Results:**

We included 206 inpatients with a major depressive episode (mean age =48.4 ± 18.6 years; males = 38.8%), of whom 155 diagnosed with MDD and 51 with BD. AD was present in 137 participants (66.5%). Mixed features (p=0.049), higher YMRS scores (p=0.004), psychotic features (p<0.05), and a diagnosis of MDD (p<0.05) were found to be associated with AD in the multiple logistic regression analysis.

**Conclusions:**

Notwithstanding some limitations, such as the cross-sectional design and the inclusion of inpatient only, our study highlights the association of AD with mixed and psychotic features, as well as with MDD. Clinical implications of these results include the possible contribution in delineating a specific symptom profile in people with AD during a major depressive episode.

**Disclosure of Interest:**

None Declared

